# Intraocular Pressure Changes Are Predictive of Ocular Hypertension Onset After Fluocinolone Acetonide Implant: Significant Cutoffs and the Role of Previous DEX Implant

**DOI:** 10.3389/fmed.2021.725349

**Published:** 2021-08-19

**Authors:** Alessandro Arrigo, Emanuela Aragona, Luigi Capone, Carlo Di Biase, Rosangela Lattanzio, Francesco Bandello

**Affiliations:** Department of Ophthalmology, IRCCS San Raffaele Scientific Institute, Vita-Salute San Raffaele University, Milan, Italy

**Keywords:** diabetic macular edema, fluocinolone acetonide implant, intraocular pressure, IOP lowering medications, trabeculectomy, dexamethasone implant

## Abstract

**Background:** Fluocinolone acetonide (FAc) implant represents a long-term strategy for the management of diabetic macular edema (DME). Because of the 3-year duration, the careful monitoring of the intraocular pressure (IOP) is necessary. The main aim of the study was to provide quantitative IOP cutoffs associated with the onset of IOP increases.

**Methods:** The study was retrospectively conducted with 2-year of follow-up. We separately considered eyes with good IOP control (Group 1), eyes requiring IOP-lowering medications (Group 2) and eyes undergoing IOP-lowering surgery (Group 3). The statistical analysis assessed Delta% IOP changes over the 2-year follow-up. ROC analysis was performed to detect significant cutoffs associated with Group 2 and Group 3. IOP changes occurring after a previously administered dexamethasone (DEX) implant were also evaluated.

**Results:** We included 48 eyes (48 patients), stratified as follows: Group 1 (25/48; 52%), Group 2 (19/48; 40%) and Group 3 (4/48; 8%). ROC analysis performed on IOP values detected 2-months later DEX implant showed a mean Delta IOP increase>24% significantly associated with IOP-lowering medications after FAc implant, whereas a mean Delta IOP increase>35% was significantly associated with IOP-lowering surgery after FAc implant. With respect to IOP changes occurred after FAc implant, our ROC analysis showed a mean Delta IOP increase>8% significantly associated with IOP-lowering medications, whereas a mean Delta IOP increase>15% was significantly associated with IOP-lowering surgery. DEX-related IOP changes showed 52% sensitivity and 100% specificity of FAc-related IOP increases.

**Conclusions:** IOP changes provides clinically relevant cutoffs associated with the onset of FAc-related IOP increases.

## Introduction

Diabetic macular edema (DME) is a common complication of diabetic retinopathy (DR), currently managed by means of intravitreal anti-VEGF injections and corticosteroids (1–3). The more recently introduced fluocinolone acetonide (FAc) 0.19 mg intravitreal drug-delivery system (ILUVIEN® Alimera Sciences, Inc., Alpharetta, GA, USA) provided a step forward regarding the long-term management of DME, guaranteeing up to 3 years of treatment duration (4–9). Although efficacy and safety have been largely demonstrated (4–9), the main issue regarding the employment of FAc implant is the management of intraocular pressure (IOP) and the prediction of IOP-related complications. Estimating the overall incidence of IOP elevation requiring lowering topical medications, the current data reported at least 20-30% of treated eyes (8–12). Furthermore, the overall incidence of uncontrolled IOP requiring IOP-lowering surgery is reported at least in the 2-5% of cases (8–12). If these numbers appear relatively small, thus making ophthalmologists confident with FAc treatment, if applied to larger number of eyes, the incidence of IOP elevations and IOP-lowering surgeries might turn out to be characterized by absolute high number of patients. Indeed, although small, IOP-related complications are remarkably higher in FAc-treated DME eyes, compared with dexamethasone (DEX) implant. It is undoubted that the IOP-related complications are more frequent in FAc-treated eyes, compared with DEX implants; taking, for example, a recent study collecting data from a high number of DEX-treated eyes, the cumulative probability of IOP increases ≥21, ≥25 and ≥35 mmHg was found equal to 50–60%, 25–30%, and 6–7% at 12–24 months, respectively, with an overall incidence of IOP-lowering surgery of 0.9% (13). In this scenario, IOP changes registered after DEX implants were found useful to try to predict IOP behavior following FAc implant (8–14), although few data are available regarding the real predictivity of DEX implant.

This study aimed to analyze in deep a selected cohort of DME eyes treated by FAc implant and reaching 2 years of follow-up, in order to (I) analyze IOP changes and reporting IOP-related complications and management; (II) establish quantitative IOP cutoffs suggestive of IOP-related events onset occurring during the follow-up; (III) establish the role of previous DEX implant in terms of IOP cutoffs predictive of complications onset; (IV) assess the predictivity of DEX-related IOP elevation on IOP increases after FAc implant.

## Materials and Methods

The study was designed as retrospective, cohort study. DME patients treated with FAc implant at the Department of Ophthalmology, IRCCS San Raffaele Scientific Institute, Milan, Italy, were included into the study. The study was approved by the local ethical committee (MIRD2020) and was conducted in accordance with Helsinki declaration. All the patients signed an informed consent before the inclusion into the study.

The inclusion criteria were eyes treated by FAc implant, followed for at least 2-years, with mandatory complete follow-up visits conducted every 2 months, with a positive history of at least one DEX implant administered before FAc implant. All the eyes were pseudophakic, accordingly with the Italian guidelines for the use of FAc implant. Exclusion criteria were the presence of media opacities, any kind of ocular surgery occurred during the 2 years of FAc follow-up, with the only exception of selective laser trabeculoplasty (SLT) or IOP-lowering surgery, any ophthalmic or systemic disease potentially affecting the results of the study (ocular hypertension, uveitis, other maculopathies or retinopathies over than DR, uncontrolled diabetes mellitus, uncontrolled systemic arterial hypertension, other endocrine system disorders). All the patients underwent complete ophthalmologic examination including ETDRS best corrected visual acuity (BCVA), slit lamp evaluation, Goldmann applanation tonometry, structural OCT (Spectralis HRA, Heidelberg Engineering; Heidelberg, Germany) with radial, raster and dense scans with high number of frames (ART > 25) and enhanced depth imaging (EDI) to highlight choroidal structures. Structural OCT images were used to extract central macular thickness (CMT) values. IOP was measured at least twice by expert ophthalmologists. During the follow-up visits, the definition of ocular hypertension was established on the IOP value > 20 mmHg. IOP was always measured in the morning for all the eyes. The criterion to start IOP lowering medication was the detection of ocular hypertension at follow-up visits. The choice of the starting drug was performed accordingly with ophthalmologists' discretion. All the eyes started with only one IOP lowering medication; combination therapy was adopted when registering poor response to monotherapy (still presence of ocular hypertension). Eyes were switched to IOP lowering surgery (trabeculectomy) for those eyes who have failed maximal medical therapy, confirmed by clinical and instrumental evaluations. Age, gender, systemic hypertension, type and duration of diabetes mellitus (DM), glycate hemoglobin (HbA1c), DR stage, previous vitrectomy, previous panretinal photocoagulation (PRP), and previous macular grid laser were considered as fixed factors. All the statistical analyses were performed by means of SPSS software package (SPSS, Illinois, USA). From the consecutive measures of IOP, we also calculated the delta values, obtained subtracting the measurements performed during the follow-up visits from the baseline values. These were used to assess the percentage changes of IOP over the 2-year follow-up and to perform the cutoff analysis. Continuous variables were assessed by unpaired *T* test. ANOVA test was used to separately analyze three subgroups, including eyes not requiring IOP-lowering medications, eyes requiring IOP-lowering medications and eyes underwent IOP-lowering surgery (trabeculectomy). Bonferroni correction was applied to assess multiple comparisons. The relationship among the considered variables was explored by Tau-Kendall correlation test. Moreover, we perform a ROC analysis to detect statistically significant IOP cutoffs, based on delta IOP values, associated with the need of IOP-lowering medications or the need of IOP-lowering surgery. ROC analysis was performed both considering IOP values registered after FAc implant and IOP values measured before and 2 months after the previous DEX implant. In case of multiple DEX implants, we considered the IOP measured after the first DEX implant. The predictive value of DEX implant was tested reporting the true positive and the false positive cases of DEX-related IOP increases followed by FAc-related IOP increases. Statistical significance was set at *p* < 0.05.

## Results

One-hundred and thirteen eyes of 79 DME patients were considered. Forty-two out of 113 eyes (37%) required IOP-lowering medications, whereas 12/113 eyes (11%) underwent IOP-lowering surgery.

Among all these patients, 48 eyes of 48 patients (25 males; mean age 68 ± 8 years) met the inclusion criteria and were considered for the analyses. The clinical data are extensively reported in Table 1. Considering that the mean IOP significantly increased during the first year of follow-up since FAc implant (*p* < 0.01), 23 out of 48 eyes (48%) required IOP-lowering medications, whereas 4/48 eyes (8%) underwent IOP-lowering surgery. The mean time from the first IOP-lowering medication was 10 ± 8 months, whereas the mean time from IOP-lowering surgery was 12 ± 2 months. The cohort of DME eyes experienced statistically significant improvements both of LogMAR BCVA (*p* = 0.02) and CMT (*p* < 0.01) during the first year of follow-up, maintained during the second year of follow-up (both *p* > 0.05).

**Table 1:** Clinical and Imaging data.

**Parameter**	**Mean ± STD**	***P*-value (baseline vs. 24 m)**
Age (years)	68 ± 8	
Gender (M/F)	25/23	
IOP Increase (%)	23/48 (48%)	
Trabeculectomy (%)	4/48 (8%)	
DM Type (1/2)	19/29 (40%/60%)	
Duration of DM (months)	25 ± 14 (36 ± 9 for DMT1 and 18 ± 10 for DMT2)	
Arterial hypertension	30/48 (63%)	
Panretinal photocoagulation	28/48 (58%)	
Focal grid macular laser	24/48 (50%)	
Vitrectomy	8/48 (17%)	
DR type (NPDR/PDR)	29/19 (60%/40%)	
HbA1c (%)	7.1 ± 0.9	
baseline_IOP (mmHg)	14 ± 2	*p* < 0.01
12m_IOP (mmHg)	17 ± 4	
24m_IOP (mmHg)	16 ± 4	
baseline_LogMAR BCVA	0.55 ± 0.39	*p* = 0.02
12m_LogMAR BCVA	0.48 ± 0.37	
24m_LogMAR BCVA	0.47 ± 0.36	
baseline_CMT (μm)	529 ± 212	*p* < 0.01
12m_CMT (μm)	338 ± 114	
24m_CMT (μm)	323 ± 130	

*IOP, Intraocular pressure; DM, diabetes mellitus; DR, diabetic retinopathy; NPDR, non-proliferative diabetic retinopathy; PDR, proliferative diabetic retinopathy; BCVA, best-corrected visual acuity; CMT, central macular thickness*.

We stratified the cohort of eyes as follows: Group 1 (not requiring IOP-lowering medications; 25/48 eyes; 52%), Group 2 (requiring IOP-lowering medications; 19/48 eyes; 40%) and Group 3 (requiring IOP-lowering surgery; 4/48 eyes; 8%). As reported in Table 2, clinical features, previous history, Baseline LogMAR BCVA and CMT values were similar among the three groups (all *p* >0.05). Group 1 and Group 2 experienced statistically significant BCVA improvement (both *p* < 0.05) during the first year of follow-up, maintaining similar values during the second year (both *p* > 0.05). On the other side, Group 3 showed stable BCVA values both during the first and the second year of follow-up (both *p* > 0.05). All the three Groups underwent significant CMT improvement during the first year of follow-up (all *p* < 0.01), keeping CMT stable at 2-year follow-up (all *p* > 0.05).

**Table 2:** Clinical and imaging data of the subgroup analysis.

**Parameter**	**Group (Mean ± STD)**			***P*** **-values**		
	**No IOP MED (1)**	**IOP lowering MED (2)**	**IOP-lowering SURG (3)**	**1 vs. 2**	**1 vs. 3**	**2 vs. 3**
Number of eyes	25 (52%)	19 (40%)	4 (8%)			
Age (years)	70 ± 8	67 ± 8	66 ± 7	all *p* > 0.05		
Gender (M/F)	12/13	9/10	4/0			
DM type (1/2)	8/17	9/10	2/2			
Duration of DM (months)	21 ± 12	30 ± 16	29 ± 11			
Arterial hypertension	15/25 (57%)	11/19 (60%)	4/4 (100%)			
Panretinal photocoagulation	13/25 (48%)	11/19 (60%)	4/4 (100%)			
Focal grid macular laser	13/25 (52%)	8/19 (40%)	3/4 (75%)			
Vitrectomy	4/25 (14%)	4/19 (21%)	0/4 (0%)			
DR Type (NPDR/PDR)	17/8 (66%/34%)	11/8 (60%/40%)	1/3 (25%/75%)			
HbA1c (%)	7.1 ± 1.1	7.2 ± 0.7	7.1 ± 0.7			
baseline_LogMAR BCVA	0.66 ± 0.42	0.43 ± 0.34	0.47 ± 0.34			
2m_LogMAR BCVA	0.56 ± 0.41	0.36 ± 0.25	0.49 ± 0.33			
4m_LogMAR BCVA	0.56 ± 0.41	0.35 ± 0.25	0.50 ± 0.32			
6m_LogMAR BCVA	0.56 ± 0.41	0.34 ± 0.25	0.45 ± 0.30			
8m_LogMAR BCVA	0.56 ± 0.41	0.34 ± 0.27	0.44 ± 0.31			
10m_LogMAR BCVA	0.57 ± 0.45	0.33 ± 0.25	0.43 ± 0.31			
12m_LogMAR BCVA	0.59 ± 0.43	0.34 ± 0.24	0.43 ± 0.31			
14m_LogMAR BCVA	0.58 ± 0.42	0.32 ± 0.26	0.43 ± 0.31			
16m_LogMAR BCVA	0.59 ± 0.44	0.32 ± 0.26	0.42 ± 0.32			
18m_LogMAR BCVA	0.56 ± 0.37	0.30 ± 0.27	0.50 ± 0.35			
20m_LogMAR BCVA	0.59 ± 0.40	0.32 ± 0.27	0.50 ± 0.35			
22m_LogMAR BCVA	0.56 ± 0.37	0.37 ± 0.26	0.50 ± 0.35			
24m_LogMAR BCVA	0.58 ± 0.39	0.32 ± 0.27	0.50 ± 0.35			
*P*-value BCVA baseline vs. 12m	<0.05	<0.05	>0.05			
*P*-value BCVA 12m vs. 24m	>0.05	>0.05	>0.05			
baseline_CMT (μm)	582 ± 266	479 ± 134	465 ± 91	all *p* > 0.05		
2m_CMT (μm)	422 ± 179	351 ± 127	314 ± 82			
4m_CMT (μm)	425 ± 207	326 ± 110	311 ± 91			
6m_CMT (μm)	387 ± 162	322 ± 88	317 ± 100			
8m_CMT (μm)	393 ± 182	330 ± 117	330 ± 121			
10m_CMT (μm)	357 ± 115	330 ± 125	318 ± 100			
12m_CMT (μm)	338 ± 92	336 ± 134	341 ± 148			
14m_CMT (μm)	349 ± 133	321 ± 115	353 ± 125			
16m_CMT (μm)	330 ± 107	324 ± 123	304 ± 111			
18m_CMT (μm)	333 ± 122	320 ± 127	303 ± 110			
20m_CMT (μm)	354 ± 151	320 ± 118	289 ± 96			
22m_CMT (μm)	322 ± 125	312 ± 114	291 ± 92			
24m_CMT (μm)	337 ± 146	314 ± 122	293 ± 92			
*P*-value CMT baseline vs. 12m	<0.01	<0.01	<0.01			
*P*-value CMT 12m vs. 24m	>0.05	>0.05	>0.05			

*IOP, intraocular pressure; DM, diabetes mellitus; DR, diabetic retinopathy; NPDR, non-proliferative diabetic retinopathy; PDR, proliferative diabetic retinopathy; BCVA, best-corrected visual acuity; CMT, central macular thickness*.

Looking at the previous IOP changes detected 2 months later the first DEX implant (Table 3), Group 1 showed stable IOP values (*p* > 0.05), with a mean Delta IOP of 4%; Group 2 and Group 3 showed statistically significant IOP increases (both *p* < 0.01), with Delta IOP values of 42 and 59%, respectively. Group 3 overall showed the highest IOP increase after DEX implant (*p* < 0.01). In all the cases, the IOP came back to normal values, thus not requiring IOP-lowering medications after the four months of DEX implant.

**Table 3:** Intraocular pressure (IOP) values in the subgroup analysis.

**Parameter**	**Group (Mean ± STD)**			***P*** **-values**		
	**No IOP-lowering MED (1)**	**IOP-lowering MED (2)**	**IOP-lowering SURG (3)**	**1 vs. 2**	**1 vs. 3**	**2 vs. 3**
IOP pre-DEX (mmHg)	15 ± 2	13 ± 1	13 ± 3	>0.05	>0.05	>0.05
IOP post-DEX (mmHg)	15 ± 2	19 ± 2	21 ± 3	<0.01*	<0.01*	<0.01*
Mean Delta IOP (%)	4	42	59	<0.01*	<0.01*	<0.01*
*P*-value pre-DEX vs. post-DEX	>0.05	<0.01	<0.01			
Baseline_IOP (mmHg)	14 ± 2	15 ± 1	13 ± 2	>0.05	>0.05	>0.05
2m_IOP (mmHg)	15 ± 2	17 ± 2	18 ± 3	<0.01*	<0.01*	>0.05
4m_IOP (mmHg)	14 ± 2	17 ± 4	20 ± 9	>0.05	<0.01*	>0.05
6m_IOP (mmHg)	15 ± 2	18 ± 3	18 ± 7	>0.05	>0.05	>0.05
8m_IOP (mmHg)	15 ± 2	17 ± 3	17 ± 4	<0.01*	<0.01*	>0.05
10m_IOP (mmHg)	15 ± 2	17 ± 3	21 ± 8	>0.05	<0.01*	<0.01*
12m_IOP (mmHg)	15 ± 2	18 ± 4	19 ± 6	>0.05	>0.05	>0.05
14m_IOP (mmHg)	15 ± 3	17 ± 5	17 ± 5	>0.05	>0.05	>0.05
16m_IOP (mmHg)	15 ± 3	16 ± 3	20 ± 7	>0.05	0.02*	0.02*
18m_IOP (mmHg)	15 ± 3	15 ± 3	23 ± 10	>0.05	<0.01*	<0.01*
20m_IOP (mmHg)	16 ± 3	16 ± 4	16 ± 5	>0.05	>0.05	>0.05
22m_IOP (mmHg)	15 ± 3	15 ± 3	20 ± 4	>0.05	<0.01*	<0.01*
24m_IOP (mmHg)	15 ± 3	16 ± 3	20 ± 4	>0.05	<0.01*	<0.01*
*P*-value baseline vs. 12m	>0.05	<0.01*	<0.01*			
*P*-value 12m vs. 24m	>0.05	<0.01*	>0.05			
Mean Delta IOP 2m (%)	3	11	37	>0.05	<0.01*	<0.01*
Mean Delta IOP 4m (%)	2	12	62	>0.05	<0.01*	<0.01*
Mean Delta IOP 6m (%)	4	21	44	<0.05*	<0.01*	<0.01*
Mean Delta IOP 8m (%)	7	12	66	>0.05	<0.01*	<0.01*
Mean Delta IOP 10m (%)	7	10	65	>0.05	<0.01*	<0.01*
Mean Delta IOP 12m (%)	7	17	49	<0.05*	<0.01*	<0.01*
Mean Delta IOP 14m (%)	8	17	59	<0.05*	<0.01*	<0.01*
Mean Delta IOP 16m (%)	9	7	55	>0.05	<0.01*	<0.01*
Mean Delta IOP 18m (%)	8	2	80	>0.05	<0.01*	<0.01*
Mean Delta IOP 20m (%)	10	6	22	>0.05	<0.01*	<0.01*
Mean Delta IOP 22m (%)	9	3	53	>0.05	<0.01*	<0.01*
Mean Delta IOP 24m (%)	8	6	55	>0.05	<0.01*	<0.01*

IOP, intraocular pressure; DEX, dexamethasone implant. Statistically significant values are marked by asterisks (*).

The three Groups started with similar IOP values before FAc implant (all *p* > 0.05). Statistically significant IOP increases were registered already at the 2-month follow-up in Group 2 and Group 3 (both *p* < 0.01). Group 1 maintained IOP values within the normal range for the entire 2-year follow-up, reaching the highest but not significant mean Delta IOP values of 12% at month 20 (*p* > 0.05). With respect to Group 2 and Group 3, although the absolute IOP value was significantly different from month 10 (17 vs. 21 mmHg; *p* < 0.01), mean Delta IOP was significantly different already at month 2 (11 vs. 37%; *p* < 0.01). The course of IOP values is extensively reported in Table 3. At the end of the follow-up, Group 2 was characterized by absolute IOP values within the normal range (16 ± 3 mmHg; *p* > 0.05) and mean Delta IOP of 6%, with respect to baseline. On the other side, Group 3 showed absolute values included in the upper margin of normal range (20 ± 4 mmHg; p>0.05) and maintaining remarkably higher mean Delta IOP (55%) with respect to baseline.

We found statistically significant correlations between Delta IOP detected after DEX implant and the need both of IOP-lowering medications (Tau-Kendall coeff. 0.738; *p* < 0.01) and IOP-lowering surgery (Tau-Kendall coeff. 0.416; *p* < 0.01), as well as between absolute IOP values after DEX implant and the need both of IOP-lowering medications (Tau-Kendall coeff. 0.658; *p* < 0.01) and IOP-lowering surgery (Tau-Kendall coeff. 0.395; *p* < 0.01) (Table 4). Furthermore, both IOP-lowering medications and IOP-lowering surgery significantly correlated with IOP values detected at month 2 (Tau-Kendall coeff. 0.457 and 0.334, respectively; *p* < 0.01) and month 4 (Tau-Kendall coeff. 0.423 and 0.384, respectively; *p* < 0.01). IOP-lowering medications significantly correlated also with IOP values measured at month 6 (Tau-Kendall coeff. 0.402; *p* < 0.01), whereas IOP-lowering surgery significantly correlated with IOP values detected at month 8 (Tau-Kendall coeff. 0.327; *p* < 0.01) and month 10 (Tau-Kendall coeff. 0.337; *p* < 0.01) (Table 4). No statistically significant correlations were found between IOP values and both LogMAR BCVA and CMT changes over the entire follow-up (both considering absolute values and Delta% variations) (all *p* > 0.05). As expected, LogMAR BCVA and CMT showed statistically significant correlations over the follow-up (cumulative Tau-Kendall coeff. 0.350; *p* < 0.01). In addition, IOP, BCVA and CMT were not influenced by previous vitrectomy, previous PRP or focal laser, HbA1c values, DR or DM type, systemic health status (all *p* > 0.05). All the considered variables were not influenced by the age (*p* > 0.05).

**Table 4:** Correlation analysis.

Delta IOP% post-DEX	IOP-lowering MED	IOP-lowering SURG		
	0.738	0.416		
	<0.01	<0.01		
IOP post-DEX	IOP-lowering MED	IOP-lowering SURG		
	0.658	0.395		
	<0.01	<0.01		
IOP-lowering MED	2m_IOP	4m_IOP	6m_IOP	
	0.457	0.423	0.402	
	<0.01	<0.01	<0.01	
IOP-lowering SURG	Delta% IOP 2m	Delta% IOP 4m	Delta% IOP 8m	Delta% IOP 10m
	0.334	0.384	0.327	0.337
	<0.01	<0.01	<0.01	<0.01

*IOP, intraocular pressure; DEX, dexamethasone implant*.

**Figure 1:**
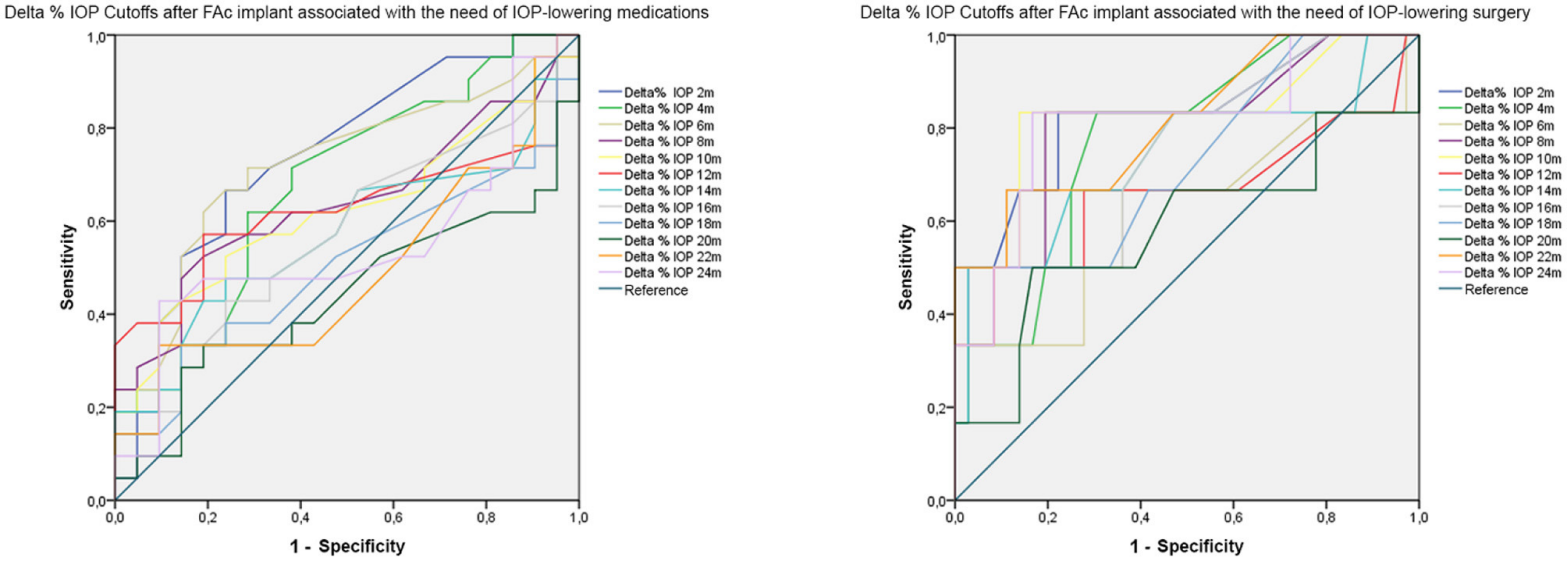
ROC analysis of Delta% IOP cutoffs associated with the need of IOP-lowering medications or IOP-lowering surgery after FAc implant.

The results of the ROC analysis are extensively reported in Table 5. In detail, we found that a mean Delta IOP increase >24% (sensitivity 0.95; specificity 0.95; *p* < 0.01) detected 2 months later DEX implant was significantly associated with the need of IOP-lowering medications after FAc implant. Furthermore, a mean Delta IOP increase >35% (sensitivity 0.83; specificity 0.73; *p* < 0.01) after 2 months from DEX implant was significantly associated with the need of IOP-lowering surgery after FAc implant. If considering the mean Delta IOP changes detected after FAc implant, 2-month (>8%; *p* < 0.01) and 6-month (>10%; *p* < 0.01) values were significantly associated with the need of IOP-lowering medications. Moreover, in terms of mean Delta IOP changes detected after FAc implant associated with the need of IOP-lowering surgery, we found statistically significant cutoff values at 2-month (>15%; *p* < 0.01), 4-month (>13%; *p* < 0.01), 8-month (>18%; *p* < 0.01) and 10-month (>18%; *p* < 0.01) examinations (Table 5). ROC curves are shown in Figure 1.

**Table 5:** ROC analysis detecting statistically significant IOP cutoffs associated with the need of IOP-lowering medications or IOP-lowering surgery after FAc implant.

**Cutoff**		**ROC value (%)**	**Sensitivity**	**Specificity**	***P*-value**
Delta IOP% post-DEX associated with IOP lowering medications after FAc implant		24.04	0.95	0.94	<0.01
		26.79	0.91	0.95	
		30.95	0.86	0.94	
Delta IOP% post-DEX associated with IOP-lowering surgery after FAc implant		30.95	0.96	0.67	<0.01
		34.52	0.83	0.73	
		37.09	0.83	0.75	
Delta IOP% after FAc implant associated with IOP lowering medications	Delta% IOP 2m	6.46	0.71	0.67	<0.01
		7.5	0.67	0.71	
		10.41	0.67	0.76	
	Delta% IOP 6m	6.9	0.71	0.67	<0.01
		9.82	0.71	0.71	
		12.92	0.67	0.71	
Delta IOP% after FAc implant associated with IOP-lowering surgery	Delta% IOP 2m	13.81	0.83	0.75	<0.01
		14.84	0.83	0.78	
		16.03	0.67	0.78	
	Delta% IOP 4m	9.82	0.83	0.62	<0.01
		12.92	0.83	0.70	
		14.36	0.67	0.75	
	Delta% IOP 8m	15.48	0.83	0.73	<0.01
		17.71	0.83	0.78	
		19.38	0.83	0.81	
	Delta% IOP 10m	16.03	0.83	0.73	<0.01
		17.71	0.83	0.81	
		19.38	0.83	0.87	

*IOP, intraocular pressure; DEX, dexamethasone implant; FAc, fluocinolone acetonide implant*.

In terms of DEX-related IOP increase >20 mmHg predictivity of FAc-related IOP increase, overall, 12/48 eyes (25%) showed IOP increase >20 mmHg 2 months after DEX implant. Among these eyes, 12/12 (100%) of eyes were characterized by IOP increase >20 mmHg after FAc implant (9 eyes (75%) requiring IOP-lowering medications and 3 eyes (25%) requiring IOP-lowering surgery). However, 11/48 additional eyes (23%) were characterized by IOP values <20 mmHg after DEX implant, but IOP increases >20 mmHg after FAc implant (10 eyes (91%) requiring IOP-lowering medications and 1 eye (9%) requiring IOP-lowering surgery). Based on our data, DEX-related IOP changes showed the following features: sensitivity 52%; specificity 100%; Positive Predictive Value 100%; Negative Predictive Value 69%; Accuracy 77%.

## Discussion

In the present study, we analyzed IOP changes occurring after FAc implant, focusing on clinically relevant cutoffs associated with the need of IOP-lowering medications or IOP-lowering surgery. Our analyses highlighted two main information. Firstly, IOP changes detected after FAc implant may be predictive of IOP control loss requiring medications or surgery. Although our ROC analysis found more timepoints associated with statistically significant IOP cutoffs, it is worth of notice that already 2-month follow-up may be clinically useful for IOP control. Indeed, a mean Delta IOP change >8% was significantly associated with the need of IOP-lowering medications, whereas a mean Delta IOP change >15% characterized the eyes requiring IOP-lowering surgery. To the best of our knowledge, this is the first study performed considering the Delta IOP variations detected in FAc-implant setting. For this reason, the current literature is poorly useful in supporting our findings. However, our data agreed with the conclusions provided by all the previous safety assessments, which considered a careful IOP monitoring mandatory in FAc implant clinical setting (8–14).

The same methodology pointed out the effectiveness of IOP monitoring already in DEX implant setting. Hence, the second main information regarded the definition of DEX-related Delta IOP cutoffs providing useful information before FAc implant. Considering the Delta IOP changes detected 2 months later DEX implant, a mean change >24% was significantly associated with the need of IOP-lowering medications after FAc implant, whereas a mean change >35% was significantly associated with IOP-lowering surgery. Remarkably, considering the 2-month timepoint, the Delta IOP changes resulted higher after DEX implant than after FAc-implant. This phenomenon might be explained considering the different pharmacokinetic profiles of the two releasing systems, being at the maximal concentration after 2 months with DEX implant and resulting not yet in the plateau phase with FAc implant (15, 16).

Furthermore, although previous DEX implant is globally considered useful to detect cortico-responder eyes before FAc implant (8, 15), to the best of our knowledge, the definition of specific IOP cutoffs to assess DEX predictivity is not available in the current literature. In the present study, we reported that DEX-related IOP changes are predictive of FAc-related IOP increases with a sensitivity of 52% and a specificity of 100%. This means that a IOP increase after DEX implant is a reliable warning of IOP control loss after FAc implant, but DEX implant is affected by at least 50% of false negative IOP changes. The reason of this phenomenon is unclear. Based on our correlation findings, we may exclude a possible cumulative effect of FAc molecules occurring during the follow-up. In particular, we found no correlations among IOP, BCVA and CMT behaviors, thus seeming to have no significant relationships. Furthermore, we found no linear increases of IOP values over the entire follow-up in all the three subgroups of eyes. In addition, the mean time of onset of clinically relevant IOP increases is relatively long (10 ± 8 months), although showing high variability, and it is remarkably close with the mean time of loss of IOP control requiring the switching to IOP-lowering surgery (12 ± 2 months). These observations differed from what observed and described after DEX implant, where IOP increases quickly occur and are generally well-managed by topical medications. Explaining these different IOP behaviors is quite challenging. If looking at the pharmacokinetics profiles of DEX and FAc implant, DEX is characterized by higher and not fixed release of drug (saw tooth shape) with the peak concentration occurring at the first 2 months (17). Conversely, FAc implant is characterized by a constant 0.2 μg/day release, allowing to reach the steady state of concentration at month-3 (16).

Although obtained from a limited number of eyes, our data suggest that the IOP increases might be governed by other possible phenomena. Both the different pharmacokinetics and absolute concentrations of the two drugs might have an influence on the trabecular meshwork and on the regulation of aqueous production/outflow. However, we might hypothesize two different mechanisms of IOP increases. Those eyes characterized by ocular hypertension onset occurring in the first months since the FAc implant might be considered as pure cortico-responders, whereas eyes showing delayed IOP increases might be characterized by other not yet understood pathogenic sources.

The mechanisms of corticosteroid induced IOP increases are partially understood. One of the most shared hypotheses attributed the IOP elevation to a reduced aqueous outflow caused by reduced activity of the trabecular meshwork and extracellular debris accumulations (18–22). However, many other metabolic pathways have been involved in the pathogenesis of corticosteroids induced glaucoma (23), including also genetic predisposition (24), thus making this complication a multifactorial phenomenon.

Although the precise pathogenesis of IOP increase cannot be evinced by this study, our data strongly remarked that the reaching of IOP values >20 mmHg is only the last step of a more complex process already detectable after 2 months since the FAc (or DEX) implant. In this scenario, as highlighted by a recent meta-analysis, IOP monitoring is a key point in FAc setting, since the proportion of FAc-implanted eyes receiving IOP-lowering medications ranged from 7% to 46%, whereas the reported need of IOP-lowering surgery ranged from 0.3% to 9.5% of cases (25). As shown by our findings, the mere detection of IOP values > 20 mmHg is probably not sufficient to properly manage this long-term treatment. On the contrary, a definition of new IOP assessment criteria, based on the evaluation of IOP values deviation from the range considered normal for each patient, might be fundamental to plan personalized follow-up strategies and to improve FAc safety profile. For this reason, future studies should be focused on the definition of even more precise Delta IOP cutoffs, based on larger patients' cohorts.

We are aware that our study labors under several possible limitations. The first complaint may be the relatively low number of eyes and the retrospective nature of the investigation. The included eyes came from real-life settings, and underwent various treatment strategies, in accordance with ophthalmologists' discretion; these differences might exert possible influence on the clinical course of the disease after FAc implant. Furthermore, further useful information, including a deep assessment of tonometry curve, pachymetry and other more specific features of glaucoma clinical setting were not performed. In addition, we limited our observations to 2 years of follow-up, being aware about FAc therapeutic duration of 3 years. Thus, further studies should be focused on the complete assessment of IOP changes over the entire 3-year FAc follow-up.

In conclusion, our study provided quantitative IOP cutoffs resulting predicting of the need of IOP-lowering medications and IOP-lowering surgery in FAc implant setting. Patients' monitoring during the previous DEX implant was found extremely useful in terms of IOP cutoffs detection, although DEX-related IOP changes resulted highly specific but poor sensitive in predicting FAc-related IOP complications. Future studies should be conducted in order to provide new IOP monitoring criteria, thus defining new guidelines for the safe administration of FAc-implant and new strategies to prevent and to manage IOP-related complications.

## Data Availability Statement

The original contributions presented in the study are included in the article/supplementary material, further inquiries can be directed to the corresponding author/s.

## Ethics Statement

The studies involving human participants were reviewed and approved by Ethical committee of IRCCS San Raffaele Scientific Institute, Milan, Italy. The patients/participants provided their written informed consent to participate in this study.

## Author Contributions

AA and EA: study design, data acquisition, data analysis, data interpretation, and manuscript draft. LC and CD: data analysis and data acquisition. FB and RL: data interpretation, manuscript revision, and study supervision. All authors contributed to the article and approved the submitted version.

## Conflict of Interest

FB consultant for: Alcon (Fort Worth, Texas, USA), Alimera Sciences (Alpharetta, Georgia, USA), Allergan Inc (Irvine, California, USA), Farmila-Thea (Clermont-Ferrand, France), Bayer Shering-Pharma (Berlin, Germany), Bausch And Lomb (Rochester, New York, USA), Genentech (San Francisco, California, USA), Hoffmann-La-Roche (Basel, Switzerland), NovagaliPharma (Évry, France), Novartis (Basel, Switzerland), Sanofi-Aventis (Paris, France), Thrombogenics (Heverlee, Belgium), Zeiss (Dublin, USA). The remaining authors declare that the research was conducted in the absence of any commercial or financial relationships that could be construed as a potential conflict of interest.

## Publisher's Note

All claims expressed in this article are solely those of the authors and do not necessarily represent those of their affiliated organizations, or those of the publisher, the editors and the reviewers. Any product that may be evaluated in this article, or claim that may be made by its manufacturer, is not guaranteed or endorsed by the publisher.
